# Activated naïve γδ T cells accelerate deep molecular response to BCR-ABL inhibitors in patients with chronic myeloid leukemia

**DOI:** 10.1038/s41408-021-00572-7

**Published:** 2021-11-16

**Authors:** Yu-Cheng Chang, Yi-Hao Chiang, Kate Hsu, Chih-Kuang Chuang, Chen-Wei Kao, Yi-Fang Chang, Ming-Chih Chang, Ken-Hong Lim, Hung-I Cheng, Yen-Ning Hsu, Caleb G. Chen

**Affiliations:** 1 Department of Hematology, MacKay Memorial Hospital, Taipei, 10449 Taiwan; 2 Department of Medicine, MacKay Medical College, New Taipei City, 25245 Taiwan; 3 Institute of Biomedical Sciences, MacKay Medical College, New Taipei City, 25245 Taiwan; 4 Transfusion Medicine & Immunogenetics Laboratories, Mackay Memorial Hospital, New Taipei City, 25160 Taiwan; 5 MacKay Junior College of Medicine, Nursing, and Management, New Taipei, 25245 Taiwan; 6 Division of Genetics and Metabolism, Department of Medical Research, MacKay Memorial Hospital, Taipei, Taiwan; 7 Department of Medical College of Medicine, Fu-Jen Catholic University, Taipei, Taiwan; 8 Department of Hematology, GCRC Laboratory, Mackay Memorial Hospital, New Taipei City, 25160 Taiwan; 9 Institute of Molecular Medicine, National Tsing-Hua University, Hsin-Chu, Taiwan

**Keywords:** Chronic myeloid leukaemia, Immunoediting

## Abstract

Tyrosine kinase inhibitors (TKIs) that target BCR-ABL are the frontline treatments in chronic myeloid leukemia (CML). Growing evidence has shown that TKIs also enhance immunity. Since gamma-delta T (γδT) cells possess the potent anticancer capability, here we investigated the potential involvement of γδT cells in TKI treatments for CML. We characterized γδT cells isolated from chronic-phase CML patients before and during TKI treatments. γδT expression increased significantly in CML patients who achieved major molecular response (MMR) and deep molecular response (DMR). Their Vδ2 subset of γδT also expanded, and increased expression of activating molecules, namely IFN-γ, perforin, and CD107a, as well as γδT cytotoxicity. Mechanistically, TKIs augmented the efflux of isopentenyl pyrophosphate (IPP) from CML cells, which stimulated IFN-γ production and γδT expansion. Notably, the size of the IFN-γ^+^ naïve γδT population in TKI-treated CML patients was strongly correlated with their rates to reach DMR and with the duration on DMR. Statistical analysis suggests that a cutoff of 7.5% IFN-γ^+^ naïve subpopulation of γδT in CML patients could serve as a determinant for MR^4.0^ sustainability. Our results highlight γδT cells as a positive regulator for TKI responses in CML patients.

## Introduction

Human γδ T cells (γδT), accounted for only 0.5–5% of total lymphocytes in circulation [1], present cytotoxic capabilities against various cancers [2–4]. In human peripheral blood (PB), most γδT cells express variable (V) δ2 T-cell receptor (TCR) that is paired with Vγ9; the remaining γδT cells express Vδ1, Vδ3, or Vδ5, each paired with a different Vγ chain [5]. Vδ2 + T cells, as the major subpopulation of γδT, are activated uniquely by isopentenyl pyrophosphate (IPP), a product of the mevalonate pathway of isoprenoid biosynthesis [6]. Bone-strengthening aminobisphosphonate (N-BP) compounds, such as zoledronate (Zometa), inhibit farnesyl pyrophosphate synthase in the mevalonate pathway and result in IPP accumulation in cells [7, 8]. ATP-binding cassette transporter A1 (ABCA1) mediates the efflux of IPP [9]. Zometa-induced inhibition of the PI3K/AKT pathway contributes to upregulation of ABCA1. N-BPs have also been found to elicit activation and expansion of Vγ9Vδ2 T cells, and to promote the release of interferon-γ (IFN-γ) [8, 10–12]. Unlike Vδ2 + T cells, Vδ1 + T cells do not respond to IPP or N-BP [13–15]. Yet Vδ1 + T cells become potent effectors against myeloid malignancies when they are activated by leukemia cells [16, 17].

In an evaluation of pan-cancer global leukocytes,γδ T population size is highly associated with a favorable outcome from treatments of hematopoietic neoplasms and solid tumors [18]. The size of Vγ9Vδ2 T population in tumor-infiltrating lymphocytes (TIL) is positively correlated with a favorable outcome for all cancers [19]. Notably, Vγ9Vδ2 T cells are abundant in CML patients. Vγ9Vδ2 T lymphocytes exert potent cytotoxic activities against CML in vitro [20].

For leukemogenesis triggered by BCR-ABL activities, anti-CML treatments with tyrosine kinase inhibitors (TKIs), such as imatinib, nilotinib, and dasatinib, drastically improve survival rates [21–23]. Currently, for prognosis, the deep molecular response (DMR) is defined by a BCR/ABL level <0.01% (equivalent to a 4-log reduction or MR^4.0^ compared to the baseline) [21–23]. A large body of evidence indicates that some CML patients attaining DMR present long-term remission even after discontinuation of BCR-ABL inhibitors [24–26]. Studies on discontinuation of TKIs attribute this relapse-free remission of CML to individual immunity, albeit the relationship between one’s immune status and treatment responses remains unclear [27, 28]. The antileukemia immune effects depend on CML progression and treatment responses [28]. Moreover, DMR seems to be correlated with increasing numbers of natural killer cells and CD8 + T cells in the peripheral blood of CML patients [29, 30]. Yet whether circulating γδT cells could help sustain DMR in treated CML patients has not been comprehensively investigated.

In the present study, we identified a significant increase of γδT cells, and their naïve and Vδ2 + subpopulations in CML patients who achieved DMR. By T-cell phenotyping, we found a direct association between the size of IFN-γ expressing naïve γδT population and the time to reach DMR or the duration on DMR. Mechanistically, we found that down-regulation of BCR-ABL activities could promote efflux of intracellular IPP from CML cells, which consequently activated the expansion of γδT cells. By probing into the mechanism, we demonstrated that the γδT population in CML patients served as a positive regulator for treatment responses.

## Materials and methods

### Patients, controls, and samples

This study was approved by the Mackay Memorial Hospital Institutional Review Board (20MMHIS425e), and was carried out in accordance with the principles of the Declaration of Helsinki. Materials used in this study were listed in Supplementary Table 1. This study recruited 142 patients with Philadelphia chromosome (Ph)-positive CML in the chronic phase (CML-CP). The demographic data including age, gender, Sokal scores, *BCR-ABL* transcript type, TKI types, molecular responses, and outcomes were listed in Table 1. Age-matched healthy adults (HA) were included as the controls. PB samples of patients were collected multiple times for quantification of *BCR-ABL* transcripts, as previously described [31]. MMR is defined as ≥3 log reduction of the BCR-ABL product on the international scale by *BCR-ABL* RT-qPCR, and DMR or MR^4.0^ at ≥4 log reduction for at least 1 year. Pre-MMR values are the levels of *BCR-ABL* transcripts >0.1% or < 10%. All recruited patients were treated with BCR-ABL inhibitors. During the follow-up period, no patients switched or discontinued TKIs, but there might be a modification of the dose due to side effects of the TKIs. About two-thirds of patients in each group received the standard dose of imatinib (400 mg), nilotinib (600 mg), or dasatinib (100 mg), throughout their disease course. In this study, their peripheral blood mononucleated cells (PBMCs) were collected by gradient centrifugation and cryopreserved until use.

**Table 1:** Demographics of the recruited patients with CML in chronic phase.

	Healthy adults (*n* = 33)	Diagnosis (*n* = 20)	Imatinib (*n* = 27)	Nilotinib (*n* = 45)	Dasatinib (*n* = 50)
Age, median (range), years	52 (25–71)	52 (26–79)	52 (30–87)	55 (31–83)	53 (26–82)
Sex, %					
Male	40	55	62	60	60
Female	60	45	38	40	40
Sokal score, %					
Low	NA	35.0	47.6	44.4	51.1
Intermediate	NA	45.0	38.1	28.9	27.7
High	NA	20.0	14.3	26.7	21.2
Transcript type, %					
b2a2	NA	15.0	4.5	22.2	10.6
b3a2	NA	35.0	91.0	66.7	63.8
b2a2/b3a2	NA	50.0	4.5	11.1	25.6
Outcome					
5-years survival (%)	NA	NA	96.3	86.7	92.0
10-years survival (%)	NA	NA	85.2	84.4	90.0
CML related mortality (%)	NA	NA	3.7	4.4	2.0
TKI duration to DMR, median (range), month					
DMR, MR^4.0^ ≥ 4 log reduction; ≤0.01^IS^	NA	NA	53 (12–141)	39 (4–115)	25 (3–95)
Follow-up Duration, median (range), month					
DMR, MR^4.0^ ≥ 4 log reduction; ≤0.01^IS^	NA	NA	142 (33–195)	93 (33–189)	72 (19–147)
TKI therapy (n)					
Pre-MMR	NA	NA	3	3	8
MMR, ≥ 3 log reduction; ≤ 0.1^IS^	NA	NA	5	10	15
DMR, MR4.0 ≥ 4 log reduction; ≤ 0.01^IS^	NA	NA	19	32	27

*MMR* major molecular response, *DMR* deep molecular response, *TKI* tyrosine kinase inhibitor.

### Flow cytometry and reagents

To quantify γδT-cell populations in the PBMCs, 5 × 10^5^ cells were stained with various combinations of fluorophore-conjugated monoclonal antibodies (mAbs): ant-Vγδ, anti-Vδ + 1, anti-Vδ + 2, anti-CD27, anti-CD45RA, anti-CD45RO, anti-CD3, anti-CD107a. Stained cells were fixed with 4% paraformaldehyde and examined by FACSCalibur (BD Biosciences), and the data were analyzed by Cell Quest Pro software (FlowJo, LLC). A total of 50,000 lymphoid events were acquired for each sample. For staining of intracellular IFN-γ, TNF-α and perforin in γδT cells, the cells were co-cultured with untreated, or TKIs-, or zoledronate-pretreated K562 cells, in the presence of Phorbol-12- myristate-13-acetate (20 ng/ml) and 2 μg/ml of ionomycin for 4 h. γδT cells then were labeled with anti-Vγδ, anti-CD3, and antihuman IFN-γ mAbs using fix-n-perm reagents. Intracellular perforin was detected using antihuman perforin mAb. The cells were examined by FACSCalibur.

### Cell culture

K562, KU812, and KCL22 cell lines were purchased from ATCC. Primary γδT cells were isolated using magnetic bead approaches according to the manufacturer’s protocol. γδT cells were isolated (to 95% purity) by negative selection. For isolation of naïve γδT cells, naïve pan-T cells were firstly isolated through negative selection and then negatively selected again for γδT cells. To analyze the effects of TKIs on CML, imatinib (2 μM), nilotinib (2 μM), and dasatinib (100 nM), as well as specific *BCL-ABL* small interfering RNA (siRNA), were tested in vitro. All cells were cultured using RPMI-1640 media.

### Killing assay

The cytotoxicity assay was performed by flow cytometry as previously described [32], with slight modification. KU812 target cells were labeled with carboxyfluorescein succinimidyl ester (CFSE) at a final concentration of 2 μM; this discriminated target cells from the effector cells. After 4-h co-culture, the cell mixture was stained with 5 μL of 7-AAD for 15 min in the dark. Flow cytometry data were analyzed on FACSCalibur. γδT-cell cytotoxicity (%) was calculated as the percentage of the cells positive for both the CFSE and 7-AAD in total CFSE positive cells, excluding % spontaneous lysis that was estimated from the negative controls.

### Cell division assay

Isolated γδT cells or naïve γδT cells from PBMCs of healthy adults were labeled with CFSE (5 μM) for 15 min at 37 °C and then incubated at 1:1 ratio with KCL22 untreated or pretreated with *BCR-ABL* siRNA for 96 h. Proliferation was assessed by the degree of CFSE dilution in γδT cells. Flow cytometry data were analyzed on FACSCalibur.

### Cytokines and perforin release assay

Isolated γδT cells from healthy donors were co-cultured with *BCR-ABL* siRNA-, TKI-, simvastatin-treated, or untreated K562 cells at a ratio of 1:1 for 24 h. Treatment with IPP (0.5 μM) was used as a control to activate γδT cells. Supernatants then were harvested for TNF-α, IFN-γ, and perforin concentrations were measured using an enzyme-linked immunosorbent assay (ELISA) kit according to the manufacturer’s instructions.

### Statistical analysis

To compare the means between two independent groups that were not normally distributed, the nonparametric Mann-Whitney *U* test was used. If two groups were normally distributed, Student’s *t* tests were applied to test for comparison. Cumulative response rates were calculated using the cumulative incidence approach and Mentle-Cox method. For these longitudinal analyses, quantitative variables were dichotomized according to their median values. The results were shown as medians if not otherwise mentioned. The cutoff points for the most promising variables were optimized by receiver-operating characteristics (ROC) curves and the Youden index. To compare three or more independent groups, one-way analysis of variance (ANOVA) with post hoc Bonferroni’s test were used. Factors were subjected to multivariate analysis using the linear regression model. The threshold for statistical significance was defined at *p* < 0.05. GraphPad Prism 8 (GraphPad Software) and SPSS 26 (SPSS Inc.) was used for all the analyses.

## Results

### Circulating γδT and subsets increased during TKI treatments for CML

**Fig. 1:**
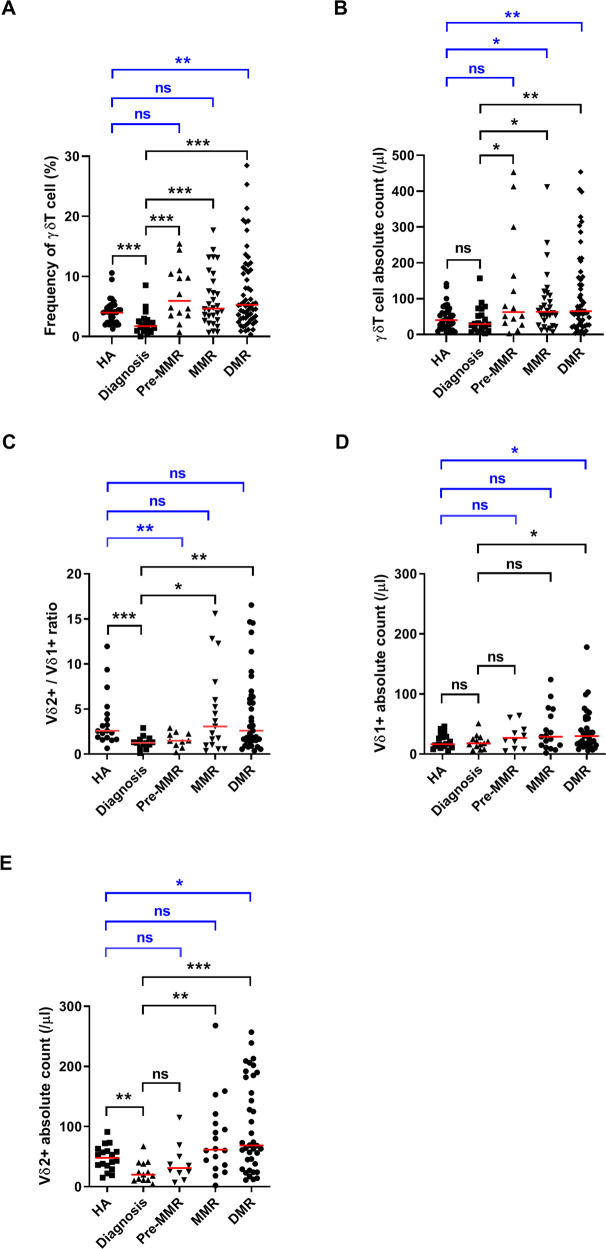
Expression of γδT cells and subsets in CML patients at diagnosis (untreated), pre-MMR, MMR, and DMR and in healthy adults (HA). **A** The percentages of TCR γδ-positive T cells were analyzed from total CD3 + T cells in the PB of CML patients and age-matched healthy adults (HA). **B** The absolute cell number in PB was enumerated per μL lymphocytes. **C** The ratio of Vδ2 to Vδ1 was calculated using the absolute number of γδT subsets. **D–E** The number of Vδ1 + and Vδ2+ T cells was measured in patients at different disease states and in healthy subjects. Statistical significance was assessed by the Mann–Whitney *U* test. Median values were indicated by the short horizontal red bars. Statistical significance was defined as *p < 0.05, **p < 0.01, and ***p < 0.001.

Immune reactivation and restoration are critical for achieving remission for CML patients [28]. We first investigated whether γδT cells could be dynamically associated with individual molecular responses in our CML cohort. Human γδT cells comprise ~5% (0.5–20%) of peripheral CD3 + cells [5]. As in Fig. 1A, before treatments, the percentage of γδT in the CD3 + population was significantly lower (1.7%) than that after TKI treatments (pre-MMR: 5.9%; MMR: 4.7%; DMR: 5.3%; *p* < 0.001 for all three groups) or that of healthy controls (HA: 4.0%). The absolute number of γδT cells also increased remarkably in the treated patients (pre-MMR: 62/μL, *p* = 0.045; MMR: 63/μL, *p* = 0.02; DMR: 65/μL, *p* = 0.005), compared to the number before treatments (29/μL) or the number of HA (40/μL) (Fig. 1). The Vδ2 + subset is predominant in the PB of healthy subjects. However, in Fig. 1C, the ratio of Vδ2/Vδ1 populations was strikingly inverted in the initially diagnosed patients (median of the ratio: 1.3), compared to healthy controls (2.6, *p* < 0.001), or treated patients on MMR (3.1, *p* = 0.034) or DMR (2.6, *p* = 0.002). These results suggest the substantial reduction of Vδ2 + T cells in the initially diagnosed, untreated patients (20/μL), compared to healthy adults (48/μL, *p* = 0.003), or patients on MMR (61/μL, *p* = 0.001) or DMR (68/μL, *p* < 0.0001) (Fig. 1E). The number of Vδ1 + T cells was similar for untreated patients (18/μL) and healthy controls (16/μL). Both Vδ1 + T (30/μL, *p* = 0.037) and Vδ2 + T (68/μL, *p* = 0.021) subsets conspicuously increased in TKI-treated patients who achieved DMR, compared to that in the healthy donors (16/μL and 48/μL, respectively) (Fig. 1D, E), suggesting persistent immune reactivity in the patients on DMR after immune recovery.

### TKIs affected expression of four γδT subpopulations in treated CML patients

**Fig. 2:**
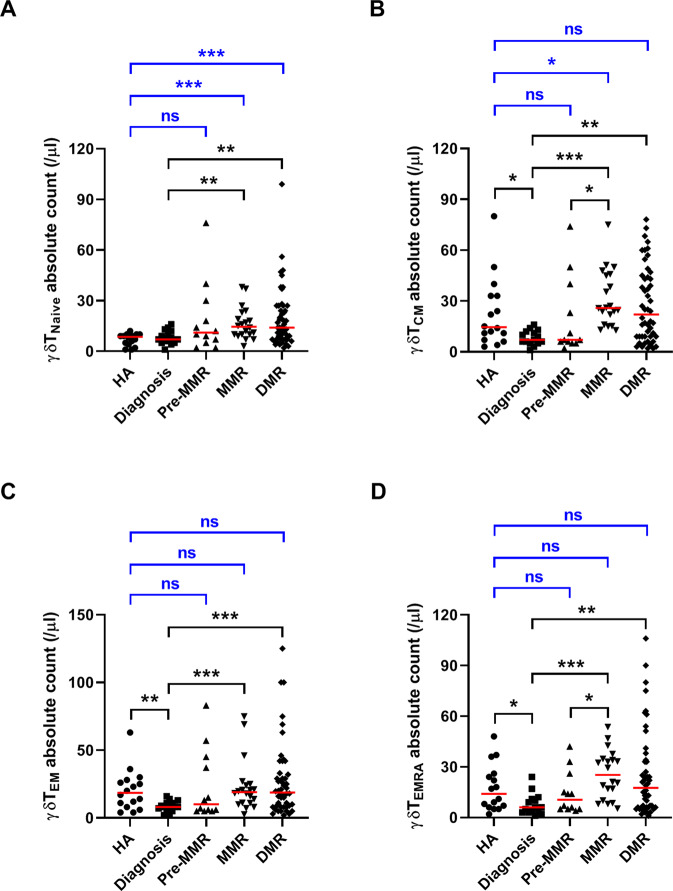
Phenotypic characterization of γδT-cell subsets. PBMCs were stained with antibodies reacting to CD3, TCR γδT, CD45RA, and CD27. Upon the analysis on gated CD3 + TCR γδT + cells, the four γδT subsets were identified as (**A**) naïve, **B** T_CM_, **C** T_EM_, and **D** T_EMRA_. The cell number of each γδT subset in PB were calculated based on the total lymphocyte count. Significant differences were found among γδT subsets isolated from healthy controls and from CML patients at different disease states. Data comparison was performed by the Student’s *t* test; if data distribution was not normally distributed, nonparametric Mann-Whitney *U* test was used. Median values were indicated by the short horizontal red bars. Statistical significance was defined as *p < 0.05, **p < 0.01, and ***p < 0.001. ns, not significant.

Based on expressions of CD27 and CD45RA, γδT cells can be categorized into naïve (CD27 + CD45RA+), central memory (T_CM_: CD27 + CD45RA-), effector memory (T_EF_: CD27-CD45RA-), and terminally differentiated effector memory (T_EMRA_: CD27-CD45RA+) subpopulations [33]. These subpopulations migrate to tumors to perform unique effector functions or to interact with CD4 + αβT cells in the secondary lymphoid tissues and trigger immune reaction [34, 35]. Expression of γδT and subsets in the initially diagnosed, untreated patients (naïve 7/μL; T_CM_: 7/μL; T_EM_: 8/μL; T_EMRA_: 6/μL) were all lower than that in healthy subjects (naïve 8/μL, T_CM_ 14/μL, T_EM_ 18/μL, and T_EMRA_ 14/μL) (Fig. 2A–D). The naïve subset accounted for 17% γδT in patients on MMR and 20% in patients on DMR, but the naïve γδT only accounted for ~7% in healthy controls. The cell number of naïve γδT cells in patients on MMR or DMR was significantly also larger than in the first-diagnosed, untreated CML patients (both *p* < 0.01) or healthy controls (both *p* < 0.001). Furthermore, these four subsets all increased in cell number after TKI treatments for CML (Supplementary Fig. 1).

For patients on DMR: their Vδ2 + T-cell subpopulation increased 3.4 folds, and their Vδ1 + T subset increased 1.7 folds. Their naïve γδT subsets expanded to significantly higher levels than that of healthy controls. This suggests that phosphoantigens like IPP might be able to stimulate Vδ2 + T expansion, particularly that of the naïve subset, in CML patients [6].

### BCR-ABL inhibitors augmented efflux of IPP from CML cells

Since nonpeptide alkylphosphates could stimulate circulating Vδ2 + T cells to proliferate and become cytotoxic [8, 10–12], we tested whether BCR-ABL inhibitors were implicated in the production of IPP in CML cells. We abrogated BCR-ABL activities in K562 cells through pharmacological and genetic inhibition in vitro. Intracellular and extracellular IPP was measured by HPLC-mass spectrometry (Supplementary Methods). The production of intracellular IPP was expedited especially in dasatinib-treated and zoledronate-treated K562 cells (Supplementary Fig. 2A). The levels of extracellular IPP elevated with BCR-ABL inhibitor treatments, which could be inhibited by ABCA1 inhibitor probucol [9] (Supplementary Fig. 2B). This observation was consistent with ABCA1 upregulation by BCR-ABL inhibitors (Supplementary Fig. 2C, D), and suggested that export of IPP could be modulated by BCR-ABL inhibitors to attenuate PI3K/AKT signaling [9].

### Co-culture of γδT and BCR-ABL-inactivated CML cells promoted γδT expansion and cytotoxicity

**Fig. 3:**
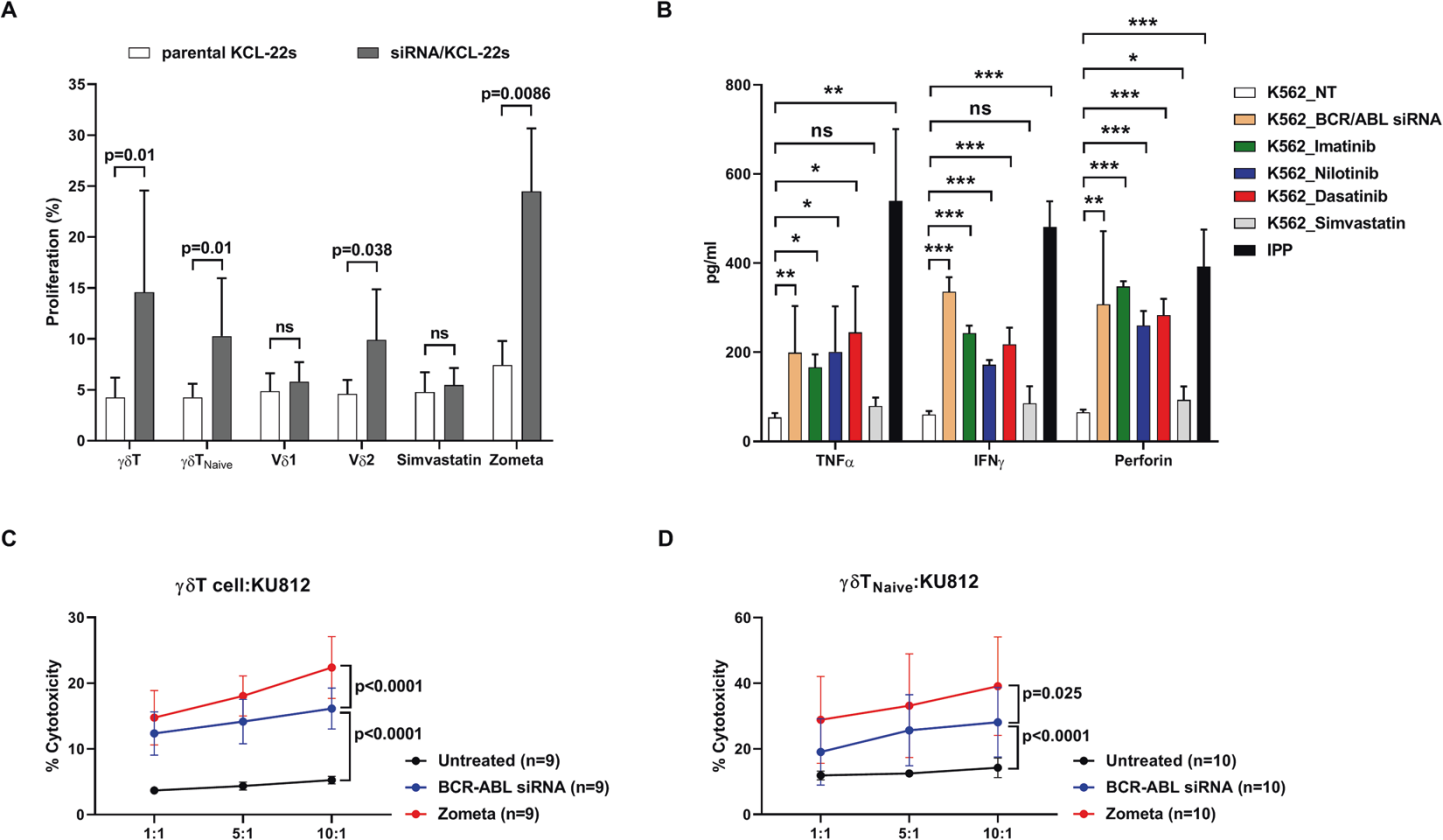
Expansion and activation of γδT and its subsets when co-cultured with CML cells treated with BCR-ABL inhibitors. **A** Expression of isolated γδT cells was determined by CFSE-labeling. CFSE-labeled γδT cells were co-cultured with *BCR-AB*L-knockdown KCL22 cells (black bar) or untreated KCL22 cells (open bar). Simvastatin-pretreated, zoledronate-pretreated, or *BCR-AB*L siRNA-pretreated KCL22 cells were used as the targets for γδT cells at a ratio of 1:1. Zoledronate (Zometa, 5 μM) but not simvastatin (100 nM) rendered KCL22 cell proliferation. **B** Expression of TNF-α, IFN-γ, and perforin release of isolated γδT cells from six healthy donors was determined by ELISA after co-culture with *BCR-ABL* siRNA-, TKI-, simvastatin-treated, or untreated K562 cells for 24 h. Treatment with IPP (0.5 μM) was used as a control to activate γδT cells. The ex-vivo killing assays were performed using isolated (**C**) γδT cells and (**D**) naïve γδT cells isolated from healthy donors. The targets could be untreated KU812 cells, or KU812 pretreated with *BCR-ABL*-specific siRNA or zoledronate. The incubation time for target-γδT cells and for target-naïve subset was 4 h and 24 h, respectively. One-way ANOVA was used for comparison between multiple groups, and paired *t* test was used to compare selected groups. Statistical significances between these groups are marked with asterisk. Data were presented as mean values ± SD. Data comparison was performed by the paired *t* test. Statistical significance was defined by *p < 0.05, **p < 0.01, and ***p < 0.001.

Because BCR-ABL inhibitor-treated CML cells released more IPP (Supplementary Fig. 2B), we postulated that their co-culture with γδT could promote γδT proliferation. Indeed, γδT co-cultured with *BCR-ABL*-knockdown (KD) KCL22 cells substantially boosted γδT proliferation (14.6%, *p* = 0.01), compared to γδT co-cultured with untreated KCL22 cells (4.2%) (Fig. 3A). Co-culture with *BCR-ABL*-KD KCL22 cells similarly promoted naïve γδT expansion (10.4% [*BCR-ABL*-KD KCL22] vs 4.3% [untreated KCL22], *p* = 0.01). Moreover, simvastatin, which blocks isoprenoid biosynthesis, abrogated the effect of *BCR-ABL* knockdown on γδT expansion. In contrast, zoledronate treatments resulted in intracellular accumulation of IPP. We further analyzed γδT by its Vδ2 + and Vδ1 + subsets: Vδ2 + proliferated more than the Vδ1 + subset when co-culture with *BCR-ABL*-KD KCL22 cells (Fig. 3A). Given that N-BPs stimulate γδT proliferation and IFN-γ production through enhancement of IPP release [8, 10–12], we next explored whether TKIs-treated CML cells could induce expression of cytotoxic cytokines, namely TNF-α and IFN-γ, and release of perforin by γδT cells. As in Fig. 3B, γδT cells co-cultured with BCR-ABL-inactivated K562 cells produced and released significantly more cytotoxicity-related cytokines. Likewise, γδT cells co-cultured with BCR-ABL-inactivated K562 cells increased the release of perforin. For verification, we co-cultured isolated γδT and the naïve subsets (Fig. 3C, D) to examine the killing effects. γδT co-cultured with either *BCR-ABL* KD or zoledronate-treated CML cells expedited cell lysis (both *p* < 0.0001). From these in-vitro results, BCR-ABL-inactivated CML cells stimulated γδT expansion and cytotoxicity via IPP, although zoledronate was more potent than BCR-ABL inhibitors.

### Circulating γδT cells in patients achieving DMR expressed high cytotoxicity-related molecules and cytotoxic functions

**Fig. 4:**
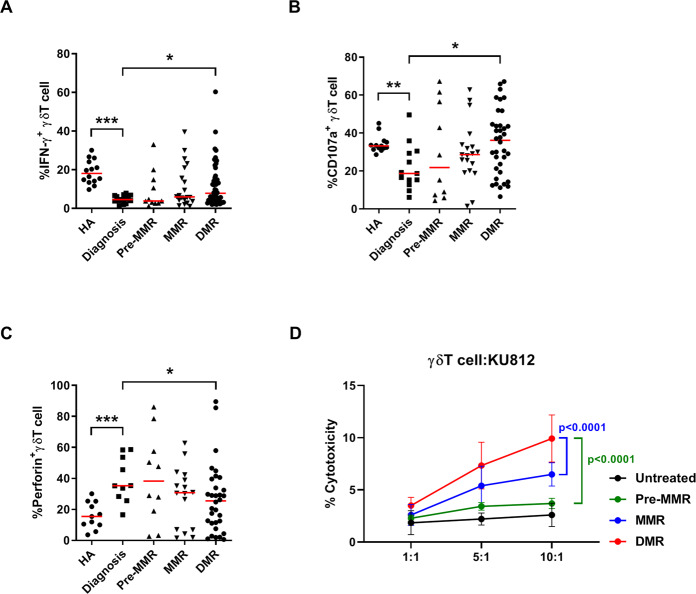
γδT cells isolated from TKI-treated CML patients showed broad reactivities to BCR-ABL-inactivated CML cells. The percentages of γδT cells expressing **A** intracellular IFN-γ, **B** perforin, and **C** surface CD107a were determined using flow cytometric analysis. Red bars denote the median. Data comparison was performed by the Mann-Whitney *U* test and paired *t* test. **D** The cytolytic activity of γδT cells isolated from patients on pre-MMR, MMR, and DMR was performed. KU812 cells were untreated or treated with *BCR-ABL*-specific siRNA and co-cultured with γδT at the indicated effector-to-target (E:T) ratios. One-way ANOVA was used for comparison between groups, and Bonferroni’s post hoc test was used to compare selected pairs. Statistically significant differences between the groups were marked with asterisks. Data were presented in mean ± SD of three patients. Statistical significance was defined by *p < 0.05, **p < 0.01, and ***p < 0.001.

We next investigated whether antileukemia effectors were enhanced in CML patients during TKI treatments. In Fig. 4A, intracellular IFN-γ^+^ γδT cells significantly increased in patients on DMR, compared to the newly diagnosed, untreated patients (8.2% [DMR] vs 4.6% [untreated], *p* = 0.012). To assess the degree of γδT degranulation, we labeled them with CD107a, a surrogate marker for degranulation, and found much higher CD107a^+^IFN-γ^+^ γδT levels in patients on DMR than in the untreated patients (36.2% [DMR] vs 18.7% [untreated], *p* = 0.035) (Fig. 4B). Consistently, the percentage of γδT cells expressing perforin diminished in patients on DMR, compared to that in untreated patients (25.5% [DMR] vs 35.3% [untreated], *p* = 0.041) (Fig. 4C). We also determined whether isolated γδT cells from the patients on pre-MMR, MMR, and DMR could exhibit similar cytotoxicity when they were co-cultured with CML cells (Fig. 4D). We observed an improved leukemia-killing ability of γδT cells isolated from CML patients with better molecular responses, particularly from patients achieving DMR (DMR to pre-MMR *p* < 0.0001; DMR to MMR *p* < 0.0001). Thus, γδT cells in CML patients on DMR expressed significantly more cytotoxic molecules, concordant with the findings from the KU812-cell killing assays (Fig. 3C, D).

### TKI treatments for CML influenced IFN-γ expression in γδT and affected molecular responses

**Fig. 5:**
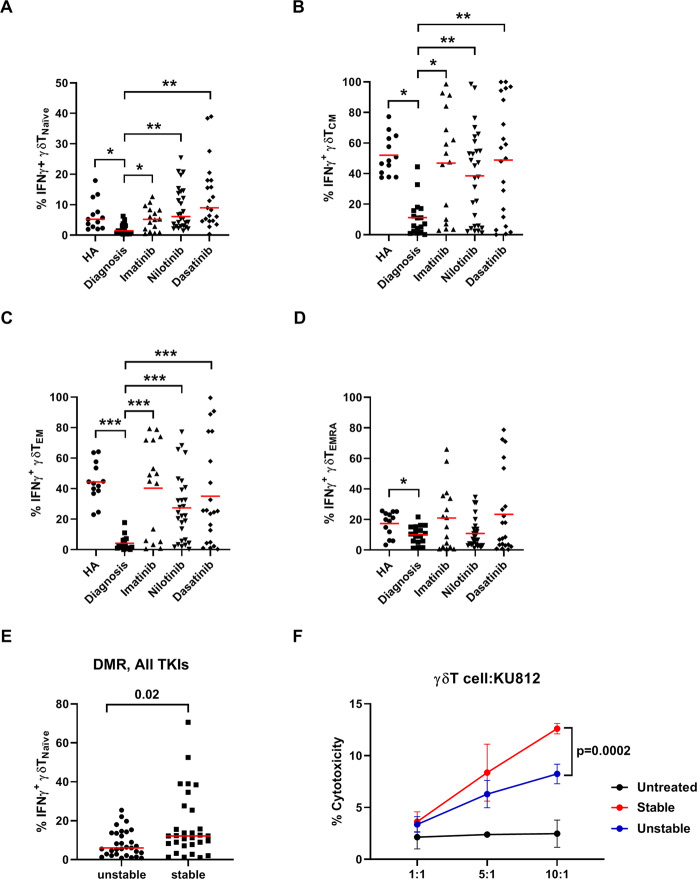
IFN-γ expressing γδT phenotypes and cytotoxicity in CML patients on DMR. The four γδT subsets were identified by cell surface CD45RA and CD27. The percentages of IFN-γ in the total γδT cells isolated from patients and healthy controls were compared among (**A**) naïve, **B** T_CM_, **C** T_EM_, and (**D**) T_EMRA_ γδT subsets. **E** Different proportions of IFN-γ + naïve γδT cells were observed between stable (sustained MR^4.0^) and unstable patients. **F** Cytotoxicity assays for isolated γδT cells from unstable and stable groups. One-way ANOVA was used for comparison between multiple groups, and Bonferroni’s post hoc test was used to compare selected pairs. Statistically significant differences between the groups are marked with asterisks. Data comparison was performed by the Mann–Whitney *U* test in dot plots. Statistical significance was defined by *p < 0.05, **p < 0.01, and ***p < 0.001.

Because of the significant increase of IFN-γ^+^ γδT cells in CML patients who reached DMR (Fig. 4A), we investigated the effects of TKIs on IFN-γ production from different γδT subsets. As in Fig. 5A–D, the fractions of the IFN-γ^+^ γδT naïve, T_CM_, and T_EM_ subsets in TKI-treated patients on DMR were substantially higher than the fractions in untreated patients. IFN-γ^+^ γδT naïve cells substantially increased by imatinib treatments (1703 cells/mL), nilotinib (3447 cells/mL), and dasatinib (4199 cells/mL) (Supplementary Fig. 1). In contrast, the percentages of IFN-γ^+^ γδT in the T_EMRA_ subset were similar for untreated and TKI-treated patients. The CML patients who were on DMR with two or more nonconsecutive loss of MR^4.0^ throughout the follow-up were considered unstable durability of MR^4.0^. The percentages of IFN-γ^+^ naïve γδT cells were remarkably larger in stable than unstable patients (*p* = 0.02) (Fig. 5E). The effector function of γδT against leukemia improved in stable patients, compared to unstable patients (*p* = 0.0002) (Fig. 5F). We also compared how IFN-γ^+^ naïve γδT cells affected molecular responses in different TKI treatments, and analyzed by grouping patient subjects into those who achieved MMR in 12 months or not [36], and those who achieved MR^4.0^ in 36 months or not [37] (Table 2). In successfully dasatinib- or nilotinib-treated patients, their IFN-γ^+^ naïve γδT was significantly higher than the unsuccessfully-treated patients. In contrast, this γδT subset in successfully imatinib-treated patients likely played a minor role.

**Table 2:** Percentage of IFNγ^+^ naïve γδT cells (Mean ± SD) in CML patients treated with TKIs.

		MMR by 12 mon		DMR by 36 mon	
		Mean ± SD	*P* value	Mean ± SD	*P* value
Imatinib	Yes	3.2 ± 2.4	0.089	5.0 ± 4.3	0.536
	No	6.3 ± 4.1		5.8 ± 3.6	
Nilotinib	Yes	11.6 ± 7.3	0.334	12.5 ± 7.5	0.015
	No	8.6 ± 6.0		6.0 ± 5.1	
Dasatinib	Yes	17.2 ± 12.2	0.085	18.6 ± 11.7	0.003
	No	8.6 ± 5.7		6.3 ± 5.9	

*MMR* major molecular response, *DMR* deep molecular response, *mon* months.

### IFN-γ+ naïve γδT as an indicator for the rate of recovery to DMR and for the durability of MR4.0

**Fig. 6:**
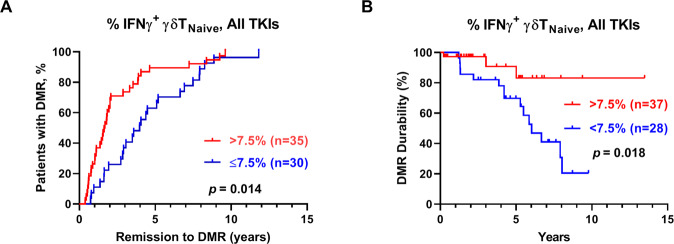
γδT-cell activation with IFN-γ expression shortening time to DMR and sustaining DMR durability. **A** Patients were dichotomized to low and high naïve γδT cells IFN-γ secretion groups. The cumulative rate to reach DMR was correlated with the fraction of IFN-γ + naïve γδT cells (7.5% as the optimal cutoff, according to the receiver-operating characteristic (ROC) curve and the Youden index analyses (AUROC 0.6720; 95% CI: 0.5346–0.8095). **B** The cumulative rate of DMR durability was associated with the fraction of IFN-γ + naïve γδT cells > 7.5%. *P*-values are calculated using the cumulative incidence approach and the Mentle-Cox method.

We next used the receiver-operating characteristic (ROC) curve and the Youden index to calculate correlations between %IFN-γ^+^ γδT and recovery to DMR (speed and duration) for CML patients. Using the cutoff of 7.5% IFN-γ^+^ naïve γδT in total γδT cells, we found that 86% of the CML patients with >7.5% IFN-γ^+^ naïve γδT achieved MR^4.0^ in 5 years, and only 63% of the CML patients with ≤7.5% of IFN-γ^+^ naïve γδT cells reached DMR in 5 years. Expression of IFN-γ^+^ naïve γδT cells also predicted the length of time in DMR, or the durability of DMR. To examine the potential predicative values of IFN-γ^+^ naïve γδT, we included predictive factors, such as age, gender, Sokal scores, *BCR-ABL* transcript type, TKI types, as well as the IFN-γ^+^ naïve γδT subset, in univariate and multivariate analyses [38, 39] (Supplementary Table 2). By univariate analysis, expression levels of IFN-γ^+^ naïve γδT (*p* = 0.004) and choices of the second-generation TKIs (*p* = 0.022) both significantly predicted DMR. By multivariate analysis, only IFN-γ^+^ naïve γδT significantly predicted DMR (odds ratio = 1.72, *p* = 0.015). Thus, the complete molecular response was more sustainable in the patients with >7.5% IFN-γ^+^ naïve γδT, compared to the patients with ≤7.5% IFN-γ^+^ naïve γδT (Fig. 6B: *p* = 0.018, Mentle-Cox test) (Fig. 6B).

## Discussion

This study explored how BCR-ABL inhibition was coordinated with γδT immunomodulation in CML treatments. Here we found that TKI-treated CML cells in vitro increased the release of IPP. Human γδT cells strongly respond to IPP, and expand for tumor lysis [8, 11, 12]. Intracellular IPP effluxes from γδT through ABCA1 transporter, and this process is up-regulated by PI3K and mTOR inhibitors [9]. Consistently, in stable CML patients, TKI treatments significantly increased circulating γδT-cell number. Importantly, the number of IFN-γ^+^ naïve γδT cells in treated CML patients was strongly associated with the time needed to achieve DMR and the durability of MR^4.0^ in following years.

In line with the findings by Rohon et al. [40]., we did not find differences in the total γδT-cell number between initially diagnosed, untreated CML patients and healthy controls. But before treatments, the Vδ2 + T count in CML patients was half that in healthy subjects. The poor Vδ2 + expression in the untreated patients rebounded to healthy levels after successful TKI treatments. The Vδ2 + γδT naïve cells are more sensitive to IPP, and respond by cell proliferation, pro-inflammatory cytokine production, and differentiation toward T_CM_ cells [14, 15, 41]. Hughes *et al*. also reported that maximal restoration of immunity in CML patients who achieved MR^4.5^ is associated with responses of effector NK cells and T cells and is reverse of immunosuppression [42].

This study revealed an association between γδT functional recovery and CML treatment responses. Though sustained DMR may be a primer for TKI discontinuation [36], a significant number of TKI-treated CML patients fail to sustain DMR and suggests that DMR alone cannot fully support TKI discontinuation. By examining how TKIs affected IFN-γ production in different γδT subsets isolated from patients on DMR (Fig. 5), we found that their IFN-γ γδT subsets were restored to similar levels as that of healthy subjects. The second-generation TKI nilotinib and dasatinib were superior to imatinib in stimulating IFN-γ production in naïve and T_EM_ γδT cells from CML patients (Fig. 5A, c). In terms of the immune effects of TKIs in CML patients, treatments with imatinib, dasatinib, or nilotinib can reduce the expression of immune suppressors including regulatory T cells (Treg) and myeloid-derived suppressor cells (MDSC) [43–46]. Imatinib treatments for CML have been found to shift immune responses to T_H_1 by increasing IFN-γ^+^ T cells [47] and cytotoxic NK cells [48]. Dasatinib inhibits a broad spectrum of kinases, such as Src, Tec, and Syk family kinases involved in innate and adaptive immune responses [40, 49, 50]. This unique activity of dasatinib induces expansion of large granular lymphocytes (LGL), including T cell or NK cell populations, and leads to favorable clinical outcomes for CML patients, whereas other TKIs do not elicit such responses [51–53]. Interestingly, ~90% LGL expansion by dasatinib was the γδT population in dasatinib-treated patients [52]. In concordance, our dasatinib-treated CML patients expressed most IFN-γ^+^ naïve γδT cells, compared to patients treated with the other two TKIs (Supplementary Fig. 1). The immunomodulatory effect of nilotinib is unclear [54], though nilotinib may trigger the expansion of CD4 effector T cells and Treg in a dose-dependent manner [44, 55].

Vδ2 + T cells present antigen activities after phosphoantigen stimulation [56, 57]. We indeed found that the naïve γδT subsets behaved like antigen-presenting cells (APCs) and displayed characteristic molecules in TKI-treated CML patients (data not shown). This APC-like differentiation of γδT cells likely broadens the anti-tumor effects. In conclusion, IFN-γ^+^ naïve γδT cells from TKI-treated CML patients played a role in shortening the time to reach DMR and in sustaining the duration on MR^4.0^. In the future, it is worthwhile to investigate the immune responses of the γδT cells in TKI-discontinued patients.

## Supplementary information

Supplementary Information
