# Supplementary material for: Activated naïve γδ T cells accelerate deep molecular response to BCR-ABL inhibitors in patients with chronic myeloid leukemia
Source: Blood Cancer J. 2021 Nov 16;11(11):182. doi: 10.1038/s41408-021-00572-7 (PMC8595379; doi:10.1038/s41408-021-00572-7)
Supplement: Supplementary file 1 — Supplementary Information [file 41408_2021_572_MOESM1_ESM.docx]

**Supplementary Information**

**Activated Naive γδ T cells Accelerate Deep Molecular Response to BCR-ABL inhibitors in Patients with Chronic Myeloid Leukemia**

Yu-Cheng Chang^1^, Yi-Hao Chiang^1,2^, Kate Hsu^*3,4,5^, Chih-Kuang Chuang^*6,7^, Chen-Wei Kao^8^, Yi-Fang Chang,^1,2,8^ Ming-Chih Chang,^1,2^ Ken-Hong Lim^1,2,8^, Hung-I Cheng^9^, Yen-Ning Hsu^9^, Caleb G. Chen^1,5,8,10^

**Materials and Methods**

**Supplementary Table 1.** **Resources of Materials**

**Quantification of IPP**

**Supplementary Table 2. Predictive factors of sustained DMR by univariate and multivariate analyses.**

**Supplementary Figure 1.**

**The absolute numbers of IFN-γ^+^ γδ T- cell immunophenotyping subsets in CML patients treated with different TKIs.**

**Supplementary Figure 2.**

**IPP were produced and exported from CML cells treated with BCR-ABL inhibitors**.

**Materials and Methods**

**Supplementary Table 1. Resources of Materials**

| Reagent and Resource | Source | Identifier |
| --- | --- | --- |
| Antibody | | |
| FITC anti-human CD3 antibody | BioLegend | 300406 |
| Brilliant Violet 421 anti-human CD27 antibody | BioLegend | 356418 |
| Brilliant Violet 650 anti-human CD40 antibody | BioLegend | 334338 |
| Brilliant Violet510 anti-human CD45RA antibody | BioLegend | 304142 |
| PE anti-human CD80 antibody | BD bioscience | 557227 |
| Brilliant Violet 785 anti-human CD86 antibody | BioLegend | 305442 |
| eFluor660 anti-human CD107a antibody | eBioscience | 50-1079-42 |
| APC anti-human HLA-DR antibody | BD bioscience | 559866 |
| PE anti-human IFNγ antibody | BioLegend | 506507 |
| Alexa Fluor 647 anti-human Perforin antibody | BioLegend | 308110 |
| PerCP/Cyanine5.5 anti-human TCR γ/δ antibody | BioLegend | 331224 |
| PerCP anti-human TCR Vδ1 antibody | Miltenyi Biotec | 130-120-441 |
| PerCP anti-human TCR Vδ2 antibody | BioLegend | 331410 |
| APC anti-human TNFα antibody | BioLegend | 502912 |
| Anti-ABCA1 antibody | abcam | Ab18180 |
| Chemicals, Peptides, and Recombinant Proteins | | |
| IPP | Echelon Biosciences | I-0050 |
| Probucol | Sigma Aldrich | P9672 |
| Imatinib | Cayman Chemicals | 13139 |
| Nilotinib | Cayman Chemicals | 10010422 |
| Dasatinib | Cayman Chemicals | 11498 |
| Zometa | TTY Biopharm | 0550020 |
| PMA | Sigma Aldrich | P1585 |
| Ionomycin | Sigma Aldrich | I9657 |
| Brefeldin A | BioLegend | 420601 |
| Lipofectamine 2000 | Invitrogen | 11668019 |
| 7-AAD Viability Staining Solution | BioLegend | 420404 |
| siRNA | | |
| BCR/ABL siRNA (h) | Thermo Fisher | 121289 |
| Critical Commercial Assays | | |
| TCR γ/δ^+^ T cell Isolation Kit, human | Miltenyi Biotec | 130-092-892 |
| Naïve Pan T Cell Isolation Kit, human | Miltenyi Biotec | 130-092-892 |
| CFSE Cell Division Tracker Kit | BioLegend | 423801 |
| Human TNFα ELISA Kit | abcam | ab181421 |
| Human IFNγ ELISA Kit | abcam | ab236895 |
| Human Perforin ELISA Kit | abcam | ab46068 |
| Experimental Models: Cell Lines | | |
| K562 | BCRC | 60007 |
| KU812 | BCRC | 60502 |
| KCL22-s | ATCC | CRL-3349 |
| Software and Algorithms | | |
| FCS express 6.0 | De Novo Software | N/A |
| Kaluza 1.5a | Beckman Coulter | N/A |
| GraphPad Prism 8 (Win) | [National Institutes of Health](https://zh.wikipedia.org/wiki/National_Institutes_of_Health) | N/A |

**Quantification of IPP**

Intracellular and extracellular IPP levels were measured according to Henneman *et al.*[1]. Briefly, 2 × 10^6^ K562 cells were incubated in media containing 1% FBS, and they were treated with imatinib, nilotinib, dasatinib, zoledronate, or *BCR-ABL* siRNA for 30 min, and then washed twice with PBS. Supernatants and cells were harvested after 24-h culture. Cell pellets were washed twice using with 100mM NH_4_HCO_3_ buffer and then sonicated in one mL of the ice-cold mixture solution (isopropanol: 100mM NH_4_HCO_3_) at 1:1 ratio for 5 min. Two-hundred fifty microliter of cell lysate was mixed with 0.5 mL ice-cold isopropanol/ 100mM NH_4_HCO_3_ and acetonitrile solution. After centrifugation at 14,000 × g for 5 min at 4°C, supernatants or cell lysates were transferred to glass tubes and dried under a stream of nitrogen at 40°C. The residues were then dissolved in 120 μl milliQ water and 10 μl of this solution was injected into the HPLC-MS/MS (QTRAP4000, AB SCIEX). According to the titration curve, the results are expressed as μmol of 2 × 10^6^ cells for intracellular IPP and extracellular IPP. Calibration mixtures contained different concentrations of IPP, and were used to generate calibration curves. To inhibit ATP binding cassette A1 (ABCA1) [2] , K562 cells were first treated with probucol (5 μM) for 15 min before treating with BCR-ABL inhibitors or zoledronate.

**Supplementary Table 2. Predictive factors of sustained DMR by univariate and multivariate analyses.**

| **Univariate analysis (Linear regression)** | | | | |
| --- | --- | --- | --- | --- |
|  | **DMR** | | | |
| **Variable** | **% patients** | **Odds** | **95%CI** | ***P* value** |
| Age |  |  |  |  |
| at diagnosis (years old) | 100 | 0.015 | -0.04~0.07 | 0.59 |
| Gender |  |  |  |  |
| Male | 41.5 | - | - | - |
| Female | 58.5 | 0.131 | -1.28~1.54 | 0.85 |
| Sokal score |  |  |  |  |
| Low | 56.9 | - | - | - |
| Intermediate | 24.6 | 0.09 | -1.68~1.86 | 0.92 |
| High | 18.5 | 0.54 | -1.2.3~2.31 | 0.54 |
| Type of transcript |  |  |  |  |
| b2a2/ b3a2 | 9.2 | - | - | - |
| b2a2 | 13.9 | 1.07 | -1.80~.94 | 0.46 |
| b3a2 | 76.9 | 2.36 | 0.01~4.71 | 0.06 |
| TKIs |  |  |  |  |
| 2G | 73.9 | - | - | - |
| IMA | 26.1 | 1.779 | 0.26~3.30 | 0.02 |
| IFN-γ^+^ naïve γδT |  |  |  |  |
| ≧7.5% | 58.5 | - | - | - |
| < 7.5% | 41.5 | 2.001 | 0.68~3.32 | 0.004 |
| **Multivariate analysis** | | | | |
|  | **DMR** | | | |
| **Variable** | **% patients** | **Odds** | **95%CI** | ***P* value** |
| Age at diagnosis (years old) | 100 | 0.001 | -0.05~0.06 | 0.83 |
| Gender (Female) | 58.5 | 0.124 | -1.22~1.47 | 0.85 |
| Sokal score (Intermediate) | 24.6 | -0.09 | -1.85~1.67 | 0.92 |
| Sokal score (High) | 18.5 | 0.13 | -1.56~1.81 | 0.88 |
| Transcripts type (b2a2) | 13.9 | 1.16 | -1.75~4.07 | 0.43 |
| Transcripts type (b3a2) | 76.9 | 1.74 | -0.62~4.09 | 0.15 |
| TKIs (IMA) | 26.1 | 1.21 | -0.36~2.79 | 0.13 |
| IFN-γ^+^ naïve γδT (< 7.5%) | 41.5 | 1.72 | 0.34~3.09 | 0.015 |

Abbreviation: DMR, deep molecular response; TKI, tyrosine kinase inhibitor; IMA, Imatinib; 2G, tyrosine kinase inhibitor of second generation; CI, confidence interval.

**Supplementary Fig 1. IFN-γ expressing γδT phenotypes in CML patients treated with different TKIs.** The absolute numbers of TCR γδ-positive T cells expressing IFN-γ were analyzed from total CD3+ T cells in the PB of CML patients and age-matched healthy adults (HA). PBMCs were stained with antibodies reacting to CD3, TCR γδT, CD45RA, and CD27. Upon the analysis on gated CD3+ TCR γδT+ cells, the four γδT subsets were identified as **a** naive, **b** T_CM_, **c** T_EM_, and **d** T_EMRA_. Data comparison was performed by the Student’s *t*-test; if data distribution was not normally distributed, non-parametric Mann-Whitney *U*-test was used. Median values were indicated by the short horizontal red bars. Statistically significance was defined as **p* < 0.05, ***p* < 0.01, and ****p* < 0.001. ns, not significant.


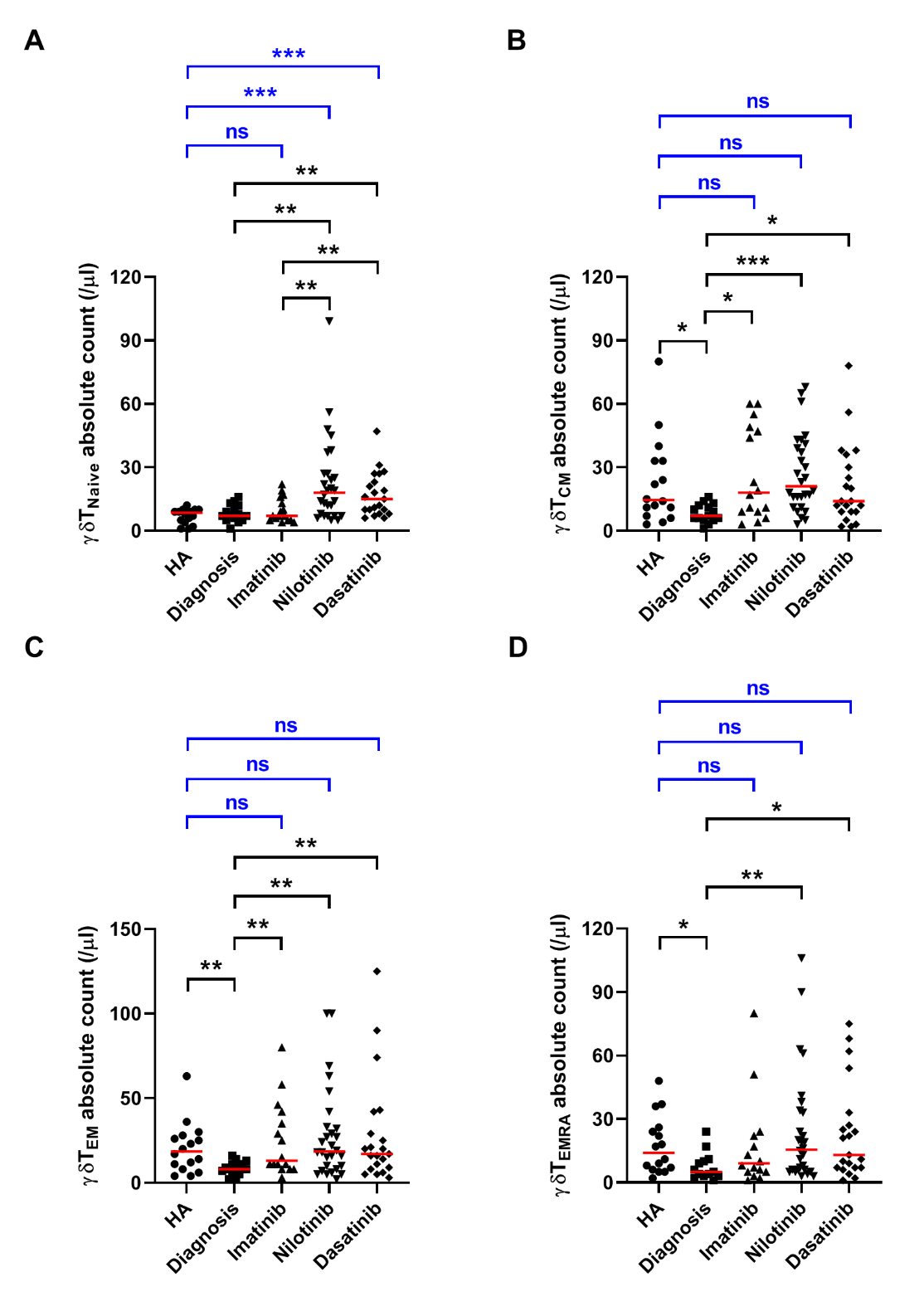


**Supplementary Fig 2.** **IPP were produced and exported from CML cells treated with BCR-ABL inhibitors**. **A-B** 2 × 10^6^ K562 cells treated with *BCR-ABL*-targeting siRNA, imatinib (2 μM), nilotinib (2 μM), dasatinib (100 nM), or zoledronate (5 μM). IPP was measured from cell pellets or supernatants. To inhibit IPP efflux, probucol (10 μM), an ABCA1 specific inhibitor, was used. **C** A representative histogram of ABCA1 expression on the surface of K562 cells with *BCR-ABL* knockdown. The numbers on the histogram indicate the geometric mean of fluorescence intensity (MFI), for ABCA1 protein. **D** K562 cells were treated with differential TKIs or *BCR-ABL*-targeting siRNA for 72 h. Zoledronate was used as a control. Bars represent the mean values ± SD from three independent experiments. Data comparison was performed by Student’s *t*-test. Statistically significance was defined by **p* < 0.05, ***p* < 0.01, and ****p* < 0.001. ns, not significant.


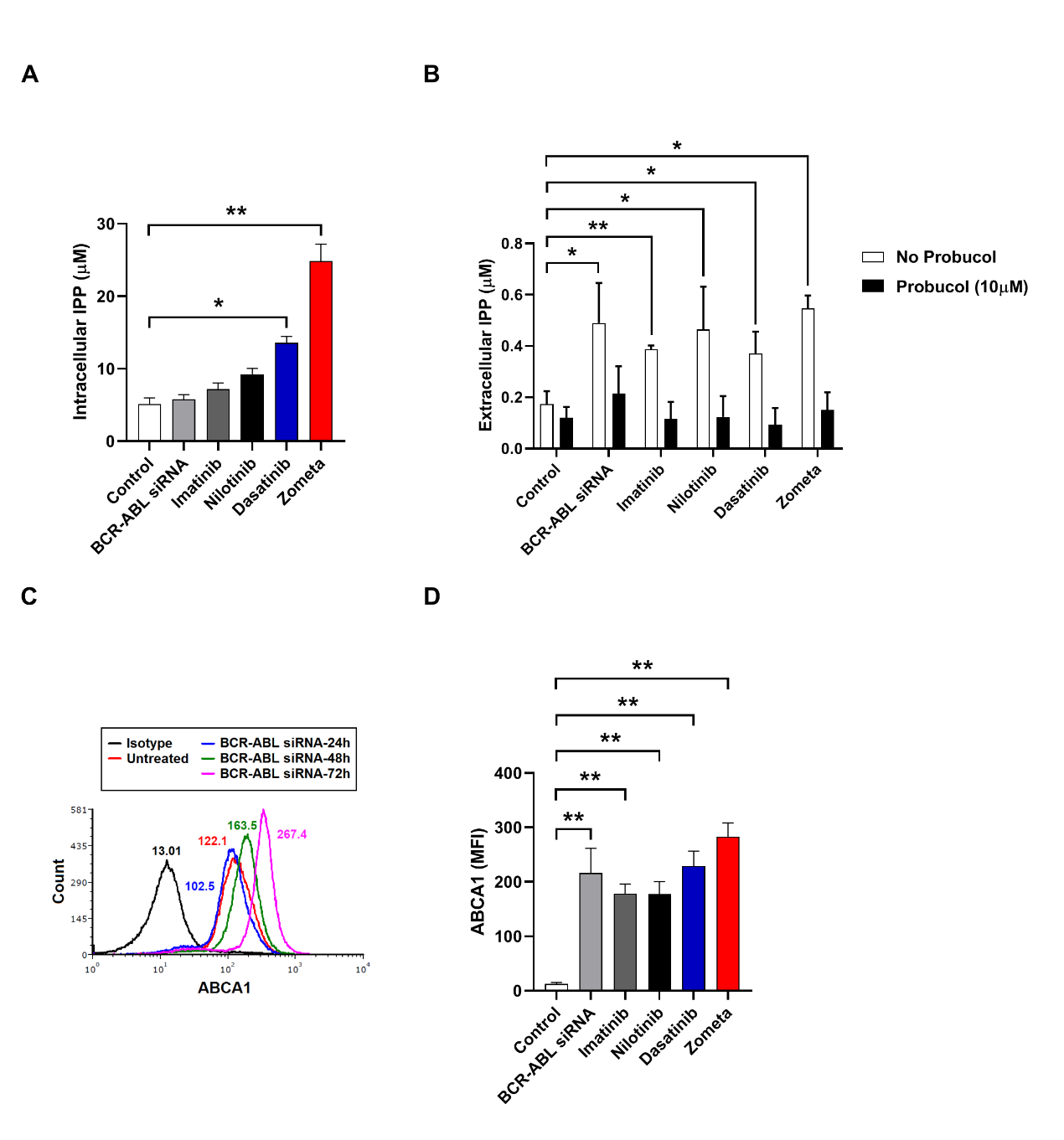


**Reference**

1. Henneman L, van Cruchten AG, Denis SW, Amolins MW, Placzek AT, Gibbs RA, et al. Detection of nonsterol isoprenoids by HPLC-MS/MS. Anal Biochem. 2008;383: 18-24.

2. Vaughan AM, Oram JF. ABCA1 redistributes membrane cholesterol independent of apolipoprotein interactions. J Lipid Res. 2003;44: 1373-80.
